# The Oxidative State of Chylomicron Remnants Influences Their Modulation of Human Monocyte Activation

**DOI:** 10.1155/2012/942512

**Published:** 2011-09-27

**Authors:** Sandra Armengol Lopez, Kathleen M. Botham, Charlotte Lawson

**Affiliations:** ^1^Department of Physiology, Faculty of Medicine and Dentistry, University of the Basque Country, Sarriena s/n, 48940 Leioa, Spain; ^2^Department of Veterinary Basic Sciences, The Royal Veterinary College, Royal College Street, London NW1 0TU, UK; ^3^Cardiovascular Biology and Inflammation Research Group, Departement of Veterinary Basic Sciences, Royal Veterinary College, Royal College Street, London NW1 0TU, UK

## Abstract

Chylomicron remnants (CMRs) contribute directly to human monocyte activation *in vitro*, by increasing reactive oxygen species (ROS) production and cell migration. In this study, the effects of the oxidative state of CMR on the degree of monocyte activation was investigated. CMR-like particles (CRLPs) were prepared in three different oxidative states, normal (CRLPs), protected from oxidation by incorporation of the antioxidant, probucol (pCRLPs), or oxidised with CuSO_4_ (oxCRLPs). Lipid accumulation and ROS production were significantly increased in primary human monocytes incubated with CRLPs, whilst secretion on monocyte chemoattractant protein-1 was reduced, but oxCRLPs had no additional effect. In contrast, pCRLPs were taken up by monocytes to a lesser extent and had no significant effect on ROS or MCP-1 secretion. These studies suggest that the oxidative state of CMRs modulates their stimulation of the activation of peripheral blood human monocytes and that dietary antioxidants may provide some protection against these atherogenic effects.

## 1. Introduction

It is now acknowledged that cardiovascular disease (CVD) is the largest killer in western countries [1]. Atherosclerosis is major cause of CVD, and it has become clear in the past decades that it is a chronic inflammatory disease [2]. Activation of monocytes is an early event in atherogenesis, triggering their adhesion to the endothelium, migration into the arterial intima, and differentiation into macrophages [3–5]. The role of low-density lipoprotein (LDL) in early atherogenesis is also well understood, it accumulates in the developing neointima where it is taken up by macrophages to form foam cells, leading to fatty streak formation, and these effects are greatly enhanced after oxidation of the particles. Oxidized LDL (oxLDL) also stimulates secretion of proinflammatory cytokines, chemokines, and other factors by macrophages, which exacerbates endothelial dysfunction and drives lesion progression [6]. In recent years, however, evidence has accumulated to indicate that lipoproteins of dietary origin are also atherogenic [7, 8].

Lipids from the diet are absorbed in the gut and secreted into lymph in large, triacylglycerol- (TG-) rich lipoproteins called chylomicrons. They pass through the thoracic duct and into the blood where they undergo rapid lipolysis, losing some of their TG to form smaller chylomicron remnants (CMRs), which then deliver the remaining TG, cholesterol, and other lipids to the liver. CMRs have been shown to enter the artery wall as efficiently as LDL and to be retained in the intima [9–11], and particles resembling CMRs have been isolated from the neointima of human atherosclerotic plaques and in animal models of atherosclerosis [12, 13]. Moreover, delayed clearance of CMRs from the blood correlates with the development of atherosclerotic lesions and is associated with consumption of western diets, obesity, and type 2 diabetes [14]. 

We and others have shown previously that CMRs are taken up and induce foam cell formation in human monocyte-derived macrophages (HMDMs) [15, 16], in macrophages derived from the human monocyte cell line THP-1 [16–18], and in the murine macrophage cell line J774 [19] and that this leads to modulation of the expression of genes involved in lipid metabolism [20]. Furthermore, we have also found that these lipoproteins influence the secretion of proinflammatory chemokines and cytokines by HMDM and THP-1 macrophages via regulation at the transcriptional level [21, 22].

Unlike LDL, CMRs are able to induce their effects on macrophage foam cell formation and on proinflammatory cytokine secretion without prior oxidation [16–22]. However, our earlier work has demonstrated that, in striking contrast to the effects of LDL oxidation, the oxidative state of CMRs is inversely related to their ability to induce foam cell formation. Thus, when CMRs are protected from oxidation by incorporation of antioxidants into the particles, lipid accumulation in macrophages is increased [17, 23], while oxidation of CMRs inhibits their ability to induce foam cell formation [18, 24]. Our studies have also shown that the oxidative state of CMRs plays an important role in their effects on proinflammatory cytokine secretion by macrophages [22]. 

Since monocytes are the precursors of macrophage foam cells, the processes which activate them and cause their recruitment to the artery wall are crucial early events in atherogenesis. Activation of monocytes during inflammation involves increased secretion of chemokines and cytokines and other vasoactive mediators together with the production of reactive oxygen species (ROS) [25–27], and recent work has suggested that CMRs may influence some of these processes. Castro Cabezas and colleagues have reported that expression of adhesion molecules is upregulated in leukocytes after a fat meal [28, 29], and studies in our laboratory have shown that CMRs are taken up by both primary human monocytes and THP-1 monocytes, causing ROS production, modulation of chemokine and cytokine secretion, and altered chemotaxis [30]. The importance of the oxidative state of CMR in their effects on monocyte activation, however, are not known.

Oxidation of CMR by the lipoxygenase and myeloperoxidase enzymes which are known to oxidize LDL is likely to occur when the particles enter and are retained in the artery wall. In addition, oxidized lipids resulting from the consumption of fat cooked at high temperatures as well as dietary lipophilic antioxidants are carried in the blood in CMR [31, 32]. It is important, therefore, to establish how the oxidative state of the particles may influence their effects on the early stages of atherogenesis. The aim of this study was to determine whether the oxidative state of CMRs influences their uptake by monocytes and subsequent pro-inflammatory pathways, using primary human monocytes and model chylomicron remnant-like particles (CRLPs).

## 2. Materials and Methods

All chemicals and tissue culture reagents were from Sigma (Poole, Dorset, UK) unless otherwise stated. Tissue culture plastics from Falcon Discovery Labware range (Fisher Scientific, UK) were used. 

### 2.1. Preparation of CRLPs

CRLPs were prepared as described previously [16]. Briefly, a lipid mixture containing 70% trilinolein, 2% cholesterol, 3% cholesteryl ester, and 25% phospholipids in 0.9% NaCl (w/v) in Tricine Buffer (20 mM, pH 7.4) was sonicated in 22–24 *μ*m for 20 min at 56°C, followed by ultracentrifugation on a stepwise density gradient at 17,000 ×g. To bind ApoE, lipid particles were collected from the top layer after ultracentrifugation and incubated with the d 1.063–1.21 g/mL fraction of human plasma (National Blood Transfusion Service, North London Centre, UK) which had previously been dialysed (18 h, 4°C). ApoE-containing-CRLPs were then isolated by two further ultracentrifugation steps in d 1.006 g/mL at 120,000 ×g, 12 h, 4°C, followed by 202,000 ×g, 4 h, 4°C, and stored at 4°C under argon until required. All preparations were used within one week. To prepare probucol-containing CRLPs (pCRLPs), 1 mg probucol was added to lipid mixture prior to sonication. CRLPs were oxidized (oxCRLPs) by incubation with CuSO_4_ (20 *μ*M) with shaking for 5 h at 37°C followed by dialysis to remove the CuSO_4_ (0.9% NaCl, 24 h, 4°C). Control preparations, obtained by a similar procedure to that described for CRLPs, but in the absence of the lipid particles, were included in all experiments to control for possible contamination factors originating from plasma. Data obtained from monocytes incubated with control preparations were similar to those derived from cells incubated in medium alone.

### 2.2. Isolation of Human Peripheral Blood Monocytes

With approval from the East London Research Ethics Committee, blood was taken by venepuncture from healthy volunteers into 15% EDTA tubes. Monocytes were isolated by negative selection using RosetteSep according to the manufacturer's instructions (StemCell Technologies, London, UK). Cells were resuspended in RPMI containing 2 mM L-glutamine, 10,000 units/mL Penicillin, 10 mg/mL streptomycin, and 10% (v/v) fetal bovine serum (PAA, Somerset, UK). Monocyte preparations were routinely stained with anti-CD14 antibody (Becton Dickinson, Oxford, UK) followed by flow cytometric analysis to verify purity.

### 2.3. Oil Red O Staining

1 × 10^6^ monocytes were incubated with CRLPs (15 *μ*g or 30 *μ*g cholesterol/mL) (or a similar volume of control preparation) and incubated at 37°C for 24 h. Cells were adhered to microscope slides by cytospin (Shandon, ThermoFisher Basingstoke, UK) and stained with Oil Red O as described previously [16]. Images were captured using a Leica upright DM4000B brightfield microscope (Leica Microsystems GmbH; Wetzlar, Germany) and the extent of staining analyzed using Volocity (Perkin Elmer, Beaconsfield, UK).

### 2.4. Measurement of Reactive Oxygen Species (ROS)

Monocytes were loaded with dihydrorhodamine-1, 2, 3 (final concentration 100 *μ*M) for 10 min at room temperature and seeded onto white opaque 96-well tissue culture plates (2.5 × 10^4^ labelled monocytes/well). CRLPs, oxCRLPs or pCRLPs containing 7.5 *μ*g/mL, 15 *μ*g/mL, or 30 *μ*g/mL cholesterol or a similar volume of control preparation were added, and plates were incubated at 37°C for up to 2 h in 5% CO_2_. Fluorescence was measured using a Wallac1410 fluorescent microtitre plate reader (Perkin Elmer, Beaconsfield, UK) at 0, 5, 10, 15, 30, and 60 min.

### 2.5. Measurement of Chemokine Secretion

Monocytes were seeded at 5 × 10^5^ cells/well in 24-well tissue culture plates, CRLPs, oxCRLPs or pCRLPs containing 15 *μ*g/mL, 30 *μ*g/mL cholesterol or a similar volume of control preparation were added and plates were incubated at 37°C/5% CO_2_ for 24 h. Cells were pelleted and the supernatants collected, snap frozen, and stored at −80°C until analysis of MCP-1 secretion using ELISA Duoset assay kits according to the manufacturer's instructions (R&D Systems, Oxford, UK).

### 2.6. Other Analytical Methods

The total cholesterol and triacylglycerol (TG) content of CRLPs was determined by enzymatic analyses (Thermo Fisher Scientific, Waltham, UK). The level of thiobarbituric acid-reacting substances (TBARS) [33] in the CRLP preparations was used to determine the extent of oxidation of the particles.

### 2.7. Statistical Analysis

Statistical analysis was carried out using Prism (Graphpad, USA). One-way ANOVA followed by Bonferroni's comparison test was used as appropriate. To analyse differences in ROS production, area under curves (AUCs) were calculated with Prism and compared by Student's *t*-test. *P* < 0.05 was considered statistically significant. All experiments were repeated with at least three separate monocyte isolates and two separate CRLP preparations.

## 3. Results and Discussion

### 3.1. Results

#### 3.1.1. Characteristics of CRLPs

The lipid and TBARS content of CRLPs, oxCRLPs, and pCRLPs is shown in Table 1. There were no significant differences in the TG and total cholesterol concentration of the three CRLP types and the TG : TC molar ratio. More importantly, the TG : TC ratio was not significantly different among the three CRLP types. Significant differences in the TBARS and lipid hydroperoxide content of the CRLPs in different oxidative states were apparent, with the values for oxCRLPs being significantly raised and those for pCRLPs significantly decreased as compared to those for CRLPs.

#### 3.1.2. Lipid Accumulation in Primary Human Monocytes after Incubation with CRLP, pCRLP, or oxCRLP

**Figure 1:**
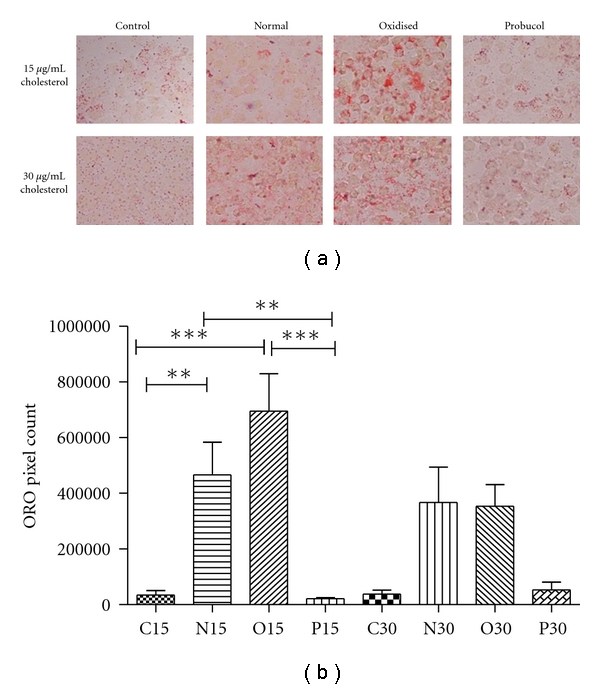
Uptake of native, oxidised, and probucol treated CRLP by primary human blood monocytes. Monocytes were incubated with CRLP at 37°C for 24 h before cytospin fixation and oil Red O staining. (a) Representative images (captured at magnification ×40 and enlarged). (b) Volocity quantitation (pixel count analysis) from *n* = 6 monocyte isolations. Approximately, 700 cells were examined for each condition. C15/C30 control equivalent volume to 15/30 *μ*g/mL cholesterol, N15/N30, CRLP at 15/30 *μ*g/mL cholesterol, O15/O30 oxCRLP at 15/30 *μ*g/mL cholesterol, P15/P30 pCRLP at 15/30 *μ*g/mL cholesterol. **P < 0.01, ***P < 0.001 analysis by one-way ANOVA and Bonferroni's posttest.

After 24 h incubation, Oil Red O staining for lipid was increased in monocytes coincubated with CRLPs or oxCRLPs, at a concentration of 15 *μ*g/mL cholesterol (Figure 1(a)), and quantitative analysis of the staining density showed that the changes were highly significant in comparison to control cells (Figure 1(b)). Uptake also appeared to be increased after incubation with the higher concentration of CRLP or oxCRLP (30 *μ*g cholesterol/mL); however, in this case the changes did not reach statistical significance. There were no significant differences in staining density between the 15 and 30 *μ*g cholesterol/mL concentrations for any of the CRLP types. In contrast, after coincubation of monocytes with pCRLPs there was no visible increase in lipid accumulation above cultures treated with control preparations, and this was confirmed by staining density analysis (Figure 1(b)). 

#### 3.1.3. Reactive Oxygen Species Generation after Incubation of Primary Human Monocytes with CRLP

**Figure 2:**
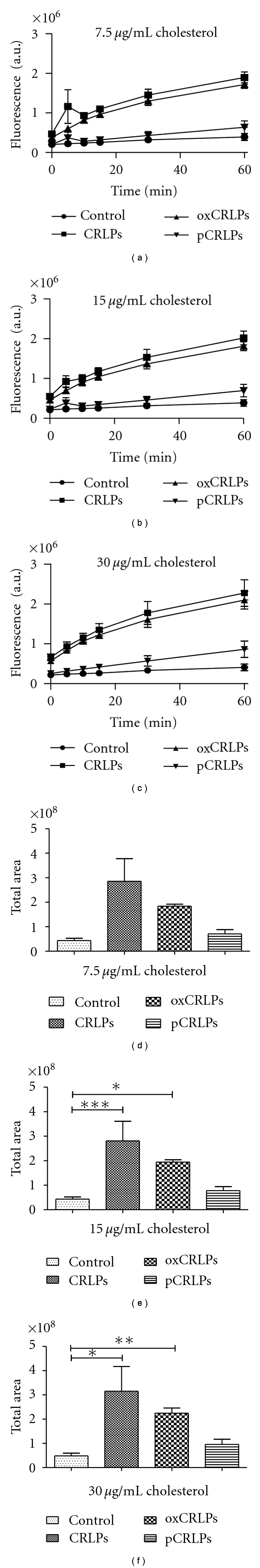
Generation of reactive oxygen species after coculture of CRLP with primary human blood monocytes. Monocytes were preloaded with dihydrorhodamine 1, 2, 3 before incubation with CRLPs, oxCRLPs, or pCRLPs and measurement of fluorescence (ROS production) at increasing timepoints. (a, b, c) Time course of ROS production (fluorescence) 7.5 *μ*g/mL, 15 *μ*g/mL and 30 *μ*g/mL cholesterol, respectively. (d, e, f) area under curve analysis (Prism) 7.5 *μ*g/mL, 15 *μ*g/mL and 30 *μ*g/mL cholesterol respectively. *n* = 5 monocyte isolations. *P < 0.05, **P < 0.01, ***P < 0.001 paired *t-*test.

To determine the effects of CRLP in different oxidative states on ROS accumulation in monocytes (Figure 2), fluorescence was measured at time points between 0 and 60 min after the addition of CRLPs, oxCRLPs, or pCRLPs to the medium (Figures 2(a)–2(c)) and the area under curve (AUC) was calculated for each experiment (Figures 2(d) and 2(e)). Addition of CRLPs or oxCRLPs led to higher ROS production as assessed by the AUC when compared to incubation with the control preparation (Figures 2(e) and 2(f)), and the changes were significant at concentrations of 15 and 30 *μ*g cholesterol/mL (Figures 2(e) and 2(f)). After incubation with pCRLP; however, there was no increase in ROS generation in comparison to control cells at any concentration of cholesterol (Figures 2(e) and 2(f)).

#### 3.1.4. MCP-1 Production by Primary Human Monocytes after Incubation with CRLP

**Figure 3:**
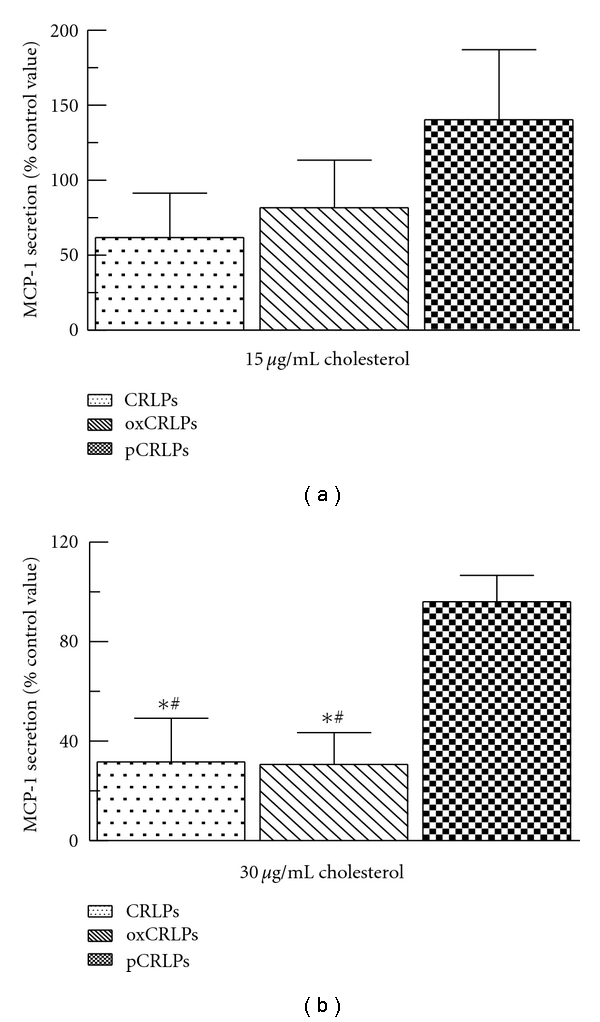
MCP-1 production by human primary blood monocytes after incubation with native, oxidised, or probucol-treated CRLPs. Monocytes were incubated with CRLPs, oxCRLPs or pCRLPs (a) 15 *μ*g/mL cholesterol; (b) 30 *μ*g/mL cholesterol) for 24 h at 37°C. After this time cells were pelleted and the supernatants were collected for analysis by ELISA. *n* = 3 monocyte isolations. *P < 0.05 compared to control, ^#^ *P* < 0.05 compared to pCRLPs-treated monocytes. One-way ANOVA with Bonferroni's posttest.

After incubation of monocytes with CRLP or oxCRLP (15 or 30 *μ*g cholesterol/mL) there was a decrease in MCP-1 secretion compared to control cells (Figure 3), and this effect reached significance at a concentration of 30 *μ*g cholesterol/mL (Figure 3(b)). Incubation of monocytes with pCRLP at similar concentrations, on the other hand, had no significant effect on MCP-1 secretion (Figure 3). 

### 3.2. Discussion

There is increasing evidence from recent *in vitro* and *in vivo* human studies in our laboratory and others to indicate that interaction of CMRs with monocytes may contribute to atherosclerosis progression. Postprandial analysis of human leukocytes isolated after ingestion of a fat meal revealed that they take up TG-rich lipoproteins, resulting in increased expression of activation markers including CD11b [28]. In addition, our previous work has demonstrated that chylomicron remnants influence proinflammatory, pro-atherogenic signalling in human primary monocytes including ROS production, cytokine and chemokine expression, as well as modulating their migration towards a chemotactic gradient [30]. This is likely to be significant for development of atherosclerosis, taken together with the finding that removal of CMR from the blood is delayed in several common conditions such as obesity and type 2 diabetes [34]. 

The oxidative state of CMR may be influenced by a number of factors. They may be protected from oxidation by the presence of lipophilic antioxidants from the diet, they are known to carry oxidized lipids from the diet and it seems likely that they are also oxidized within the artery wall by the processes which are known to cause LDL oxidation [31, 32, 35], and the relatively large and potentially polyunsaturated fatty acid-rich CMRs particles are thought to deliver a greater oxidant load to the artery wall than LDL [36]. Furthermore, our earlier work has established that the oxidative state of CMR has profound effects on their interactions with macrophages [18, 22, 24] and thus is important for their potential atherogenicity. In the present study, we have investigated the importance of the oxidative state of CRLP on monocyte lipid accumulation and activation. The model CRLPs used have been shown to resemble physiological CMR in their size, density, and lipid composition and to be metabolised in a similar way both *in vivo* and *in vitro* in cell cultures [16, 19, 37–40]. Moreover, the maximum concentration of CRLP used (30 *μ*g cholesterol/mL (78 *μ*M)) is well within the range found in triglyceride-rich lipoproteins (TRL) in human plasma (postprandially; TRL cholesterol values have been reported to reach 180–250 *μ*M [41, 42]). 

Our previous findings with macrophages using both HMDM and THP-1 macrophages have demonstrated that the effects of CRLPs on lipid accumulation are inversely related to their oxidative state of the particles [17, 18, 22]. In contrast, in primary human monocytes, CRLPs protected from oxidation by the incorporation of probucol were found to inhibit intracellular lipid accumulation (Figure 1), whilst oxCRLPs behaved in a similar manner to CRLP. Thus, prior to differentiation of monocytes into macrophages, changes in the oxidative state of CRLP have effects on intracellular lipid accumulation which more closely resemble those seen with LDL. This difference may be due to the receptor-mediated mechanisms used by the cells to take up the particles. Our previous work has suggested that the enhanced uptake of probucol-containing CRLPs by THP-1 macrophages is due to increased uptake via the LDL-receptor-related protein-1 [22]; however, the expression of this protein has been found to be low in monocytes and induced only on differentiation into macrophages [43]. Thus, reduced expression of LRP-1 in monocytes may account for both the lower uptake of pCRLP as compared to CRLP in these cells and for the differing effects of the presence of probucol on uptake of the particles in monocytes as compared to macrophages.

ROS production is stimulated in monocytes as a defence against infection and during other inflammatory reactions [3–5]. Human monocytes have been reported to generate ROS when exposed to oxidised LDL [44], and CMRs have been found to increase ROS production in THP-1 cells [30]. In agreement with our earlier study [30], CRLP treatment of monocytes caused rapid and prolonged generation of ROS (Figure 2). Although this effect was not enhanced when the particles were oxidized, it was abolished completely when pCRLPs were used. These results suggest that the presence of antioxidant in CMRs inhibits their induction of monocyte ROS production.

Human peripheral blood monocytes are known to increase the secretion of MCP-1 in response to inflammation [4]. We have found previously, however, that CRLP cause a marked decrease in MCP-1 secretion by human monocytes [30], and we have also demonstrated similar effects in macrophages using HMDMs and THP-1 cells [21, 22]. Evaluation of the effects of the oxidative state of the CRLPs in macrophages showed that oxCRLPs caused a similar reduction to that observed with CRLPs, but, when the particles were protected from oxidation with probucol, the effect was significantly reduced [22]. The results of the present study indicate that monocytes behave like macrophages in the response of MCP-1 secretion to changes in the oxidative state of CRLP, with oxCRLP and CRLP decreasing production to a similar extent, while the incorporation of antioxidant into the particles abolishes the effect (Figure 3). However, in our earlier monocyte study [30], we demonstrated that a decrease in medium concentrations of MCP-1 after exposure of monocytes to CRLP increased cell migration towards a higher concentration of the chemokine. This finding suggests that CMR may cause an increase in the chemotactic gradient of MCP-1 across the endothelium because of reduced MCP-1 secretion by monocytes in the blood vessel lumen microenvironment thus having a promigratory effect on circulating monocytes. Our present results, therefore, suggest that the presence of antioxidants in CMR may decrease their propensity to enhance monocyte chemotaxis.

Previous studies with oxLDL have demonstrated that its oxidative state is important for the reported proinflammatory, proatherogenic effects in macrophages [5, 26, 45]. Interestingly, although clinical trials with dietary supplementation of antioxidants have proved disappointing, [46] *in vitro* antioxidants have shown efficacy. Treatment of endothelial cells with the antioxidant flavonoid, luteolin, protects them from the effects of oxLDL effects by downregulation of the lectin-like oxidised LDL receptor [47]. Similarly, the citrus-derived flavonoid nobiletin has recently been reported to inhibit monocyte to macrophage differentiation and scavenger receptor activity in THP-1 cells via inhibition of PKC [48]. Although we cannot completely rule out the possibility that the effects observed in the present work are specifically due to the presence of probucol, rather than protection of the particles from oxidation, our previous work with macrophages showing that probucol and lycopene, a chemically unrelated antioxidant, have remarkably similar effects on lipid accumulation suggest that this is not the case. Thus, the present work provides evidence to suggest that antioxidants from the diet carried in CMR may help to protect against the effects of these lipoproteins in promoting the activation of human primary monocytes, but further studies are required to substantiate this conclusion.

Previous work in our laboratory has shown that downregulation of NF-*κ*B activation is involved in the inhibition of proinflammatory cytokines secretion by CRLPs [21]. It seems likely, therefore, that NF-*κ*B is involved in the modulation of monocyte activation by CMRs, and this is supported by the finding from our previous study that CRLP-stimulated monocyte ROS production is mediated via NF-*κ*B [30]. Thus, there may be early activation of this pathway followed by later inhibition of de novo NF-*κ*B synthesis, leading to downregulation of constitutive MCP-1 expression.

## 4. Conclusions

This study demonstrates that the changes in the oxidative state of CRLP influence their effects on peripheral blood monocytes. Thus, protection of the particles by the incorporation of the antioxidant probucol decreases the induction of lipid accumulation and ROS production in the cells observed with CRLP and oxCRLP but abolishes the suppressive effect on MCP-1 secretion. The results support the hypothesis that CMR play a role in the increase in monocyte activation which contributes to early atherosclerotic lesion formation and suggest that dietary antioxidants carried in CMR may reduce or prevent some of the potentially inflammatory effects of the lipoproteins on these cells.

## Figures and Tables

**Table 1:** Lipids and TBARS content of CRLP.

Parameter	CRLPs	oxCRLPs	pCRLPs
TG (*μ*mol/mL)	9.44 ± 2.33	6.01 ± 1.31	7.82 ± 1.50
TC (*μ*mol/mL)	0.98 ± 0.24	0.61 ± 0.18	1.03 ± 0.21
TG : TC (molar ratio)	10.1 ± 1.6	12.7 ± 3.1	7.5 ± 0.9
TBARS (nmol MDA/*μ*mol TG)	0.78 ± 0.05	4.00 ± 0.90**	0.39 ± 0.15**

CRLPs, oxCRLPs or pCRLPs were prepared as described in Section 2 and the TG, total cholesterol (TC) and TBARS content was measured. Data are the mean 4 preparations. Significance limits; **P < 0.01 versus CRLP.
